# Prevalence of XMRV in blood donors, HTLV and HIV cohorts

**DOI:** 10.1186/1742-4690-8-S1-A222

**Published:** 2011-06-06

**Authors:** Xiaoxing Qiu, Priscilla Swanson, Ning Tang, Gregor W Leckie, Sushil Devare, Gerald Schochetman, John Hackett

**Affiliations:** 1 Abbott Diagnostics, Infectious Diseases, Abbott Park, North Chicago, Illinois, 60064, USA; 2 Abbott Molecular Inc., Des Plaines, IL, 60018, USA

## Background

PCR-based testing has been widely utilized to assess the prevalence of Xenotropic Murine Leukemia Virus-related Virus (XMRV) in prostate cancer and chronic fatigue syndrome patients. An alternative approach, screening for antibodies elicited by XMRV infection, represents a more desirable option for large-scale epidemiologic studies. In this study, blood donor and retrovirus-infected populations were screened for serologic evidence of XMRV infection.

## Methods

Plasma from 1000 US blood donors, 100 HIV-1 infected Cameroonians, 486 HTLV-I infected Japanese, and 156 Japanese HTLV-uninfected controls were screened for antibodies to XMRV gp70 and p15E using recombinant-based chemiluminescence immunoassays (CMIAs). CMIA reactive samples were further evaluated by Western Blot (WB) and real-time RT-PCR for XMRV pol and env sequences.

## Results

Of US donors, 0.8% (8/1000) were CMIA reactive: 1 p15E and 3 of 7 gp70 reactive samples WB confirmed yielding a 0.4% seroreactive rate. No HIV-1 infected specimens were reactive. Of the Japanese samples, 1/156 uninfected (0.6%) and 20/486 HTLV-infected samples (4.1%) had detectable p15E antibodies by CMIA and WB. Eight additional HTLV-infected samples were gp70 reactive; 4 of 486 (0.8%) were gp70 reactive in WB. No XMRV pol or env sequences were detected in the seroreactives.

## Conclusions

XMRV seroprevalence ranged from 0 - 0.6% in US blood donors, HIV-1 infected and HTLV uninfected subjects. Notably, 4.1% of Japanese HTLV-I infected individuals were p15E reactive. Inspection of sequence homology between HTLV and XMRV revealed a high level of conservation within the immunodominant region of HTLV gp21 suggesting increased seroreactivity is due to cross-reactive antibodies.

